# Framing Heartaches: The Cardiac ECM and the Effects of Age

**DOI:** 10.3390/ijms24054713

**Published:** 2023-03-01

**Authors:** Nathalie Ringström, Charlotte Edling, Giovanna Nalesso, Kamalan Jeevaratnam

**Affiliations:** Faculty of Health and Medical Science, University of Surrey, Guildford GU2 7AL, UK

**Keywords:** ageing, extracellular matrix, cardiovascular medicine, cardiac remodelling

## Abstract

The cardiac extracellular matrix (ECM) is involved in several pathological conditions, and age itself is also associated with certain changes in the heart: it gets larger and stiffer, and it develops an increased risk of abnormal intrinsic rhythm. This, therefore, makes conditions such as atrial arrythmia more common. Many of these changes are directly related to the ECM, yet the proteomic composition of the ECM and how it changes with age is not fully resolved. The limited research progress in this field is mainly due to the intrinsic challenges in unravelling tightly bound cardiac proteomic components and also the time-consuming and costly dependency on animal models. This review aims to give an overview of the composition of the cardiac ECM, how different components aid the function of the healthy heart, how the ECM is remodelled and how it is affected by ageing.

## 1. Age and the Cardiac Extracellular Matrix—A Relationship That Needs More Attention

Despite rapid medical progress in most fields, ageing continues to be an unavoidable risk factor for many diseases. Ageing of the heart is associated with an array of pathological conditions such as myocardial infarction (MI), hypertension and atrial fibrillation (AF) [1,2,3]. Over the last couple of decades, cardiac molecular research has been focused on cardiomyocytes and other cells. However, more recently, it has become increasingly clear that the complex, acellular mesh surrounding the cells plays a larger role than previously thought in the development of certain pathological conditions [4,5]. Whilst relatively little is known about the cardiac extracellular matrix (ECM), it has been proven to play a role in cardiac, age-related conditions such as AF and cardiac fibrosis (CF) [4,5]. To date, mass spectrometry continues to be the gold standard in proteomic studies, but its limited dynamic range means that samples need to be enriched before analysis, e.g., through decellularisation followed by extraction of the ECM proteins [6]. As such, the question of what the cardiac ECM consists of does not yet have a comprehensive answer, and even less is known about how this proteomic landscape changes with age. This review aims to summarise what the main components of the cardiac ECM are, and how these change with age.

## 2. Components of the Cardiac ECM

**Figure 1:**
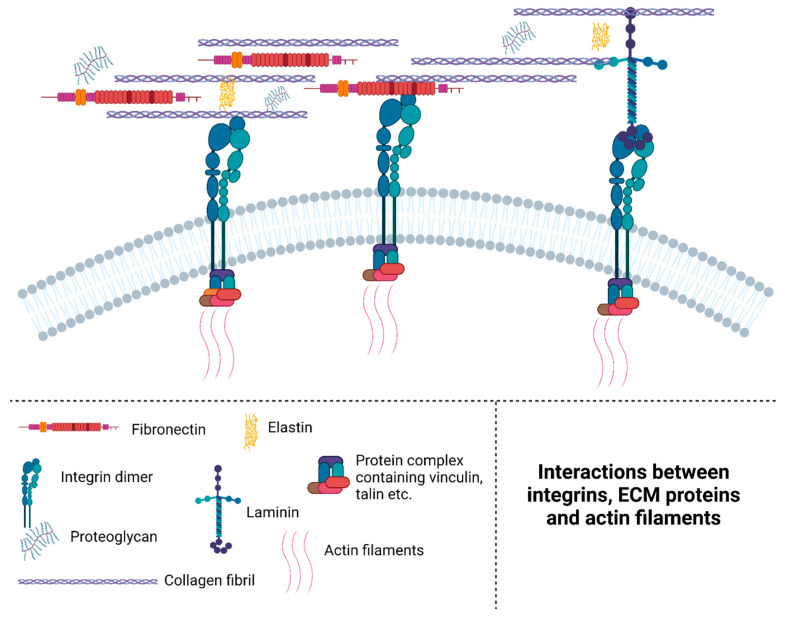
Interactions between integrins, ECM proteins and actin filaments. Integrins are important components in the anchoring of cells to the ECM, and also in the ability of the cell to sense and respond to mechanical stimuli of the environment. On the intracellular surface, integrin binds actin filament through a protein complex containing, amongst other proteins, vinculin and talin. On the extracellular surface, the integrin dimer is capable of interacting with a number of ECM proteins, such as laminin, collagen and fibronectin. Created using BioRender.com.

The ECM is a mesh consisting of a number of different proteins. It contains a large amount of heavily glycosylated proteins, such as fibronectin, and various types of collagens [7]. Glycosylation is crucial for proteins such as collagen, fibronectin and secreted protein acidic and rich in cysteine (SPARC) to fold and function properly [8,9,10]. See Figure 1 for a graphical overview of the interactions between laminin and other ECM proteins. The main components and their most important functions can be found in Table 1.

### 2.1. Epimysium, Perimysium, Endomysium and the Basement Membrane

In addition to the space between the cells in the heart, there are some structures that are extra enriched in ECM. These are the epimysium, the perimysium, the endomysium and the basement membrane (BM); thus, they will be described below. The cardiac ECM is not homogenous and uniform throughout the heart: it has different structures and different compositions depending on the location within the heart. The epimysium, perimysium and the endomysium are non-cellular, fibrous structures and compose the largest components of the cardiac ECM [11]. The epimysium is a thin sheet surrounding the whole heart, enveloping all of the chambers. In turn, the perimysium envelopes more sizeable groups of cardiomyocytes. Finally, the endomysium is the fibrous sheet that encloses individual cardiomyocytes. All three structures serve to protect the cardiomyocytes and aid the supply of nutrients by housing vasculature [11].

In addition to the endomysium, individual cardiomyocytes are also surrounded by the BM. The BM consists of two structures which resemble thin sheet-like networks. These structures are the lamina lucida and the lamina densa [12]. Each of these consists of their own unique compositions of proteins. The structure adjacent to the cardiomyocytes, the lamina lucida, is mainly composed of laminins, and the structure adjacent to the extracellular matrix, the lamina densa, is mainly composed of collagen type IV [13]. Both layers are also rich in perlecan, versican, entactin, nidogen, SPARC, hyaluronic acid, fibronectin and chondroitin sulphate proteoglycans [14]. 

Like the endomyosium, the BM also aids to protect and support the individual cardiomyocytes through aiding the overall structural integrity of the cardiac tissue [15]. Additionally, the BM aids proper alignment, polarity and organisation of the cardiomyocytes, is involved in cell migration and attachment and also plays a role in cell–cell communication [15]. It enables cells to sense mechanical stimuli such as the stiffness of the environment, and also forms a semi-permeable membrane, enabling cells to be kept separate from one another. This enhances cellular integrity whilst simultaneously facilitating the exchange of nutrients, hormones and other bioligands [15]. It aids regulation of the electrical conduits in the heart in two ways. Firstly, a correctly formed BM influences the formation of sarcomeres [16]. This is made possible through interactions between laminins and integrins with the sarcolemma [16]. In addition, the BM contains a large amount of negatively charged sialic acid which is able to bind calcium ions [17]. When this sialic acid component is removed from the cardiomyocytes in vitro, the exchange of calcium ions through the cellular membrane increases dramatically [17].

### 2.2. Glycoproteins of the Cardiac ECM

#### 2.2.1. Collagen

Collagen is the most abundant protein in the ECM of several different tissue types, including the cardiac ECM [18]. There are two main classes of collagens: fibrillar and non-fibrillar. All collagen types exist in the tissue in the form of a triple helix, also called tropocollagen [19]. The strands in this triple helix have repetitive sequences, mainly composed of glycine and proline or hydroxyproline [19]. As proline is a bulky amino acid, it grants stability to the tropocollagen molecule. Additionally, the plentiful glycine residues increase the amount of hydrogen between the individual chains in the triple helix, further aiding stability and rigidity to the molecule [19]. Together with the geometrical structure of the triple helix, it results in a protein that possesses incredible toughness, insolubility, and strength. 

In the cardiac ECM, collagen proteins are essential for anchoring cells to the ECM through interactions with integrins. They lend strength and elasticity to the heart, they play a role in several important ECM mediated cellular pathways and they are essential for normal functioning of the electrical currents in the heart [20,21]. 

In terms of mass, the fibrillar collagen is vastly more abundant than the non-fibrillar types in cardiac tissue. In fibrillar collagen, the individual tropocollagen molecules congregate with other collagen triple helices into striated fibrils. These, in turn, assemble into large fibres through the action of lysyl oxidase, an enzyme secreted by fibroblasts [19]. The crosslinking enhances the strength in the tissue but can hinder repair if not kept in check. In the adult heart, collagen type I and III are the most abundant of the fibrillar collagen types, but type V is also present. Collagen type I lends strength and resilience to tissues, whilst collagen type III enhances the flexible properties of the cardiac tissue [22]. Additionally, collagen type I is essential for proper functioning of the electrical circuits in the heart [21]. Finally, fibrillar collagen types have also been shown to play roles in cell proliferation [23].

Non-fibrillar collagen has a structure that is similar to that of fibrillar collagen. However, the triple helices do not form large fibres through lysyl oxidase mediated crosslinking [24]. In addition, the tertiary structured domains on the N- and C-terminals on the procollagen molecule are not cleaved off. Whilst less abundant than fibrillar collagen, they still play important roles in the cardiac ECM. Depending on the precise type, non-fibrillar collagens aid the anchoring of fibres in the ECM by forming mesh-like structures. They are also involved in modifying the properties of the fibrillar collagen in the tissue [25]. The most important non-fibrillar collagen types in the cardiac tissue are collagen type IV and VI. Collagen type IV is essential for the structure and function of the BM [13]. Collagen type VI is a microfibril forming collagen and forms a mesh that aids support to the fibrillar collagen type I and III in the heart [26].

#### 2.2.2. SPARC

Secreted protein acidic and rich in cysteine (SPARC), also called osteonectin or BM40, is a matricellular protein located in the ECM of a large number of different types of tissues, including cardiac tissue [8]. It has a high affinity for collagen, and it is suggested to be involved in the processing of procollagen and subsequent deposition of this [27]. Additionally, it is involved in diverse events such as differentiation, cell signalling and adhesion [28,29].

#### 2.2.3. Fibronectin

Fibronectin is a sizeable protein of 440–450 kDa, with several glycosylation sites and isomers and is produced by cardiofibroblasts, myofibroblasts and endothelial cells [30,31]. Its main function is as an adapter protein between the cells in the cardiac tissue and other proteins in the ECM such as collagen and fibrin through interactions with transmembrane integrins [32]. It has a high affinity for these ECM compounds, and as such can be considered to be “the glue” of the cardiac ECM that enables cells to adhere to the ECM network. It also functions as the frame upon which collagen is deposited [33]. In addition to this structural role, it also aids cell–cell communication in the heart. The integrins that fibronectin binds to are known to activate downstream cellular signalling via the PI3K-Akt and the MAPK pathways [34].

#### 2.2.4. Elastin

As the heart is constantly exposed to mechanical forces, it does not only require structural integrity from proteins such as collagen I, but it also needs elasticity so that it remains supple after each heartbeat. Along with collagen type III, different types of elastin are essential for this purpose. At its first stage it exists in the form of monomers called tropoelastin, which later form large polymeric molecules, the mature elastin protein [35]. As elastin is required to function like a spring of sorts, its structure is highly specific: it is a dynamic protein able to rapidly and drastically change its shape. Between contractions, the elastin in the heart is in a relaxed, highly disordered form. When it stretches out, the elastin proteins undergo rapid crosslinking. It is this crosslinking that gives stability to the stretched-out protein [36]. Elastin is highly hydrophobic, and it is the hydrophobic forces that enable it to quickly relax and return to its original form [36]. Due to these properties of elastin, it is a protein that is essential for a properly functioning heart.

#### 2.2.5. Laminin

As described above, the BM is essential for a number of different functions in the heart, and proteins in the laminin family are important components of basal lamina of the BM. All the proteins in this family are very large, several hundred kDa, and they have three different domains, the α-, β- and γ- chains [37]. These form a coil at the base of the protein, which later divides into three branches, forming the shape of a cross [37]. There are different classes of each chain and, thus, many combinations for laminin proteins. However, only 11 different laminin proteins have been found in vivo [38]. The cross-like structure of laminin is the key to its ability to connect other ECM proteins with each other, which aids the mesh-like structure of the BM and offers stability. Laminin interacts with collagen IV for which it has a high affinity, forming the base of the structure in the basal lamina [39].

#### 2.2.6. Tenascin

Tenascins are a small group of large glycoproteins which are mainly expressed during embryonic development and they tend to be upregulated during cardiac injury [40]. Tenascins interfere with the action of fibronectin, and as such, it can be seen as an anti-adhesive protein with opposite functions of fibronectin [41]. Additionally, tenascins are able to interact with fibrodermal growth factors (FGF) and TGF-β [42]. It is not clear precisely how tenascin affects cardiac remodelling. As it interacts with proteins such as TGF-β and fibronectin, and is overexpressed in ECM-related diseases such MI and myocarditis, it is clear that it formulates a potential target in this aspect and should be investigated further [40].

#### 2.2.7. Hyaluronan

Hyaluronan, also called hyaluronic acid, is the only glycosaminoglycan (GAG) in the cardiac ECM that is not bound to a core protein. It is also unique in how it is the only GAG that is not sulphated. Like all GAGs, it is able to bind large amounts of water [43]. Due to the immense water binding properties of hyaluronan, it was long considered solely as an inert molecule whose function was only to “gel” the interstitial fluid and the extracellular matrix in tissues, thus adding shock absorbing properties. Indeed, hyaluronan is essential in the cardiac ECM for this purpose, but in recent years, it has been discovered that it is also involved in processes such as wound healing, immune responses and cell proliferation and migration [44,45].

One important role of hyaluronan is its role in fibroblast proliferation and differentiation into myofibroblasts. Normally, fibroblasts differentiate into myofibroblasts upon activation of a pathway involving transforming growth factor beta (TGF-β) and SMAD, amongst other proteins. An excess of hyaluronan interacts with this pathway through the CD44- and EGFR- receptors and leads to increased differentiation of fibroblasts into myofibroblasts [46,47]. As myofibroblasts have an increased secretion of ECM-proteins, such as collagen, hyaluronan is directly linked to proper functioning of the cardiac ECM.

### 2.3. Proteoglycans—Decorin, Biglycan and Periostin

In addition to the proteins mentioned above, the cardiac ECM is rich in proteoglycans. Aggrecan, versican, decorin, biglycan and perlecan could be considered as the most important proteoglycans. As aggrecan, versican and perlecan are mainly expressed during foetal development, they will not be described here [48,49,50,51].

Decorin is an ECM protein belonging to the small leucine rich family of proteoglycans (SLRPs). It has a core protein surrounded by one chrondroitin sulphate or dermatan sulphate molecule. It is able to interact with many different types of proteins including ECM proteins such as fibronectin, collagen type I and III as well as growth factors such as TGF-β [52,53,54]. As such, it has a wide variety of functions. It is best known for its ability to modulate collagen type I, II and III by “decorating” the fibrils, hence the name decorin [54,55]. The interactions between decorin and collagen are crucial for matrix stability: decorin knock-out mice have weaker connective tissue compared to the wild type [56,57]. Furthermore, decorin affects ECM remodelling through its interactions with TGF-β. It is able to competitively bind TGF-β receptors and also bind the TGF-β ligands themselves [58,59]. As described earlier, TGF-β is a cytokine which effectively causes fibroblasts to differentiate into the highly secretory myofibroblasts. Thus, decorin is a highly potent “anti-fibrotic” protein and may be a drug target for fibrosis of a range of different tissues including the heart.

Biglycan, like decorin, is a member of the SLPR family. It has similar functions to decorin: it binds and modulates collagen fibrils and is able to bind TGF-β [58,59]. Interestingly, studies have shown that even though the two proteins are similar in terms of function, they are not interchangeable and mice lacking one cannot overcome this deficiency through an increase in the other [60].

Periostin, osteoblast-specific factor 2, is a relatively large protein that first was associated with its role in bone development, hence its name. However, during the last few decades, several studies have highlighted the roles of periostin in the ECM remodelling in several other tissues. It regulates collagen type I fibrollogenesis and is also associated with differentiation of fibroblasts into myofibroblasts [61,62]. As such, periostin is pro-fibrotic and tends to be upregulated during fibrosis.

### 2.4. Matrikines

Matrikines are a relatively new concept referring to incomplete protein fragments originating from components in the ECM [63]. They result from incomplete proteolytic cleavage and are biologically active compounds that influence processes such as cell migration and proliferation, and the composition and turn-over of the cardiac ECM. As such, they make up an important, but often overlooked, component of the cardiac ECM. Interestingly, the function of the protein before cleavage is often different to that of the matrikine. As an example, fibronectin when exposed to the right enzymes is cleaved into the matrikine anastellin or fibstatin. As previously mentioned, the functions of fibronectin include, e.g., cell adhesion and migration. However, anastellin inhibits lysophospolipid signalling and also suppresses metastasis, amongst other functions [64,65].

In addition to the cardiac ECM being a source of compounds that when cleaved in certain ways become potent and bioactive ligands in the form of matrikines, some of these matrikines are able to directly modulate the ECM from which they came. The most important example is the glycyl-L-histodyl-L-lysine (GHK) peptide. The GHK peptide is commonly associated with saliva, plasma and urine. In the cardiac tissue, it originates from the alpha chain in collagen type I [66]. When complexed with copper (II) ions, it becomes activated, and it has been shown to enhance wound healing, reduce inflammation and contribute to DNA repair [67,68,69]. When in this active form, it also stimulates production of type I collagen, GAGs, MMPs and small proteoglycans such as decorin [70]. To date, the effects of matrikines on the ECM have mainly been studied in relation to the skin and metastasis. However, it is likely that they also play similar roles in the cardiac ECM.

**Table 1:** Major structural and proteomic components of the cardiac ECM.

	Structure and Location	Functions	References
Epimysium, perimysium and endomysium	Thin, fibrous sheets. The epimysium surrounds the whole heart. The perimysium surrounds groups of cardiomyocytes. The endomysium surrounds individual cardiomyocytes.	Protection of the cardiomyocytes. Facilitates supply of nutrients by housing vasculature.	[11]
Basement membrane	Lamina lucida: Adjacent to the ECM. Consists mainly of laminins. Lamina densa: Adjacent to the cardiomyocytes. Consists mainly of collagen type IV.	Protection and support of individual cardiomyocytes. Aids cellular alignment, polarity and organisation, adhesion and migration. Enables cells to sense mechanical stimuli. Regulates electrical conduits. Facilitates cellular integrity by forming a semi-permeable membrane.	[12,13,14,15,16]
Collagen type I and III	Fibrillar collagens. Crosslinked.	Collagen type I lends strength and resilience, aids isolation of electrical currents. Collagen type III enhances flexible properties.	[19,20,21,22]
Collagen type IV and VI	Non-fibrillar collagens.	Collagen type IV is essential for the functions of the BM. Collagen type VI forms a mesh supporting collagen type I and III.	[13,24,26]
Fibronectin	Large (440–450 kDa), with several glycosylation sites and isomers.	Cellular adhesion, forms a framework which collagen is deposited upon and aids cellular communication.	[31,32,33,34]
Elastin	Disordered form in between contractions. Undergoes rapid crosslinking when stretched. Highly hydrophobic.	Aids elasticity to the heart.	[35,36]
Laminin	Large (several hundred kDa), with 3 domains forming a cross shape.	Connects proteins in the ECM and the BM; see Figure 1 for an overview of how laminin interacts with several different proteins.	[37,39]
Tenastinc	Large proteins (180–250 kDa) with several isotypes.	Mainly expressed during embryonic development and upregulated during injury. Interferes with the actions of fibronectin.	[40,41,42]
Hyaluronan	Anionic, nonsulphated glycosaminoglycan (GAG)	Adds shock absorbing properties to the ECM. Involved in wound healing, immune responses, cellular proliferation and migration. An excess of HA leads to an increased differentiation of myofibroblasts.	[44,45,46,47]
Decorin and biglycan	Core proteins surrounded by one chondroitin sulphate or dermatan sulphate.	Decorin modulates collagen type I and III by “decorating” the fibrils. These interactions between the proteins are essential for stability of the ECM. It also interacts with TGF-β through competitive binding, further affecting the ECM. Biglycan has the same functions, but studies have shown they are not interchangeable.	[52,53,54,55,56,57,58,59,60]
Periostin	Relatively large protein of ~90 kDa	Regulates collagen type I fibrillogenesis. Associated with differentiation of fibroblasts into myofibroblasts.	[61,62]
Matrikines	Varies. The result of incomplete proteolytic cleavage of different ECM proteins such as fibronectin. [63]	Varies. The function is often different from that of the original ECM protein. Glycyl-L-histodyl-L-lysine (GHK) is an important matrikine example. It is involved in stimulation of collagen type I, GAGs and MMP production.	[67,68,69,71]

## 3. Remodelling of the Cardiac ECM—The Role of Fibroblasts, MMPs and TIMPs

In physiological conditions, heart function relies on a homeostatic equilibrium between synthesis and degradation of ECM components. Cardiomyocytes, endothelial cells, macrophages and fibroblasts all participate in the turnover of ECM components. However, cardiac fibroblasts play the most important role as they produce and secrete a large part of the fibrous components of the ECM including collagen, fibronectin and hyaluronan [72]. 

During stress such as inflammation or myocardial infarction, the microenvironment in the heart will change in terms of mechanical forces and availability of bioactive molecules. Cardiac fibroblasts react to these changes and are activated into myofibroblasts through interactions with proinflammatory ligands such as transforming growth factor beta (TGF-β), interleukin 1 beta (IL-1β) and reactive oxygen species (ROS) [73,74]. Activated fibroblasts are hyper secreting compared to normal fibroblasts, thus promoting the generation of scar tissue. Whilst essential to prevent rupture of the damaged tissue and to promote scar formation, the scar tissue that this overproduction of ECM components leads to is inferior to healthy tissue in terms of cardiac functioning, described below.

Activated fibroblasts also promote the degradation of the ECM in the injured area. By increasing their production of the matrix metalloproteinases (MMPs) MMP-3 and MMP-8, activated fibroblasts help make space for the scar formation that follows [75].

During the last stages of fibrosis, the number of myofibroblasts decrease to permit the newly formed scar to mature [76].

In addition to the direct role of fibroblasts in depositing and depleting ECM components, they also appear to contribute to the hypertrophic maturation of cardiomyocytes observed in many ECM-related pathological conditions. Activated fibroblasts increase the level of collagen crosslinking, thereby increasing the stiffness of the ECM. This promotes the activation of protein kinase C (PKCα and δ) in murine cardiomyocytes, which in turn plays a role in the pathogenesis behind cardiac hypertrophy [77]. The activation of PKC occurs through activation of endothelin- and α1 adrenergic receptors, which leads to downstream effects activating PLC, RhoA and PKC, which finally leads to the transcription of genes, resulting in cardiac hypertrophy [78].

In addition to the described mechanisms of fibroblast activation, epigenetic factors and exosomes both play important roles in regulating fibroblast activity. Indeed, epigenetics has been proven to be involved in the regulation of the cardiac ECM during cardiac development and in pathological conditions such as fibrosis, as reviewed elsewhere [79,80,81].

As described above, the fibroblast is the main contributor to deposition of ECM proteins. As cardiac remodelling, during physiological and pathological conditions, includes not only the deposition of ECM components into the matrix but also the degradation of the same, the question of how the cardiac ECM is degraded when needed also requires addressing. One important part of this is the interplay between MMPs and tissue inhibitors of matrix metalloproteinases (TIMPs).

MMPs are the main proteolytic enzymes of the cardiac ECM and are able to digest ECM proteins such as collagen, elastin and various proteoglycans. There are 24 different MMP genes in humans, which code for 23 different MMPs, as 2 of these are identical [82]. In the healthy heart, none of these are expressed at high levels. However, certain pathological conditions come with an upregulation of some MMPs, causing them to degrade the ECM. For example, the increased levels of inflammatory cytokines following MI causes a large number of different MMPs, such as -2, -7 and -9, to increase in the left ventricle [83,84,85].

To add further complexity to the mechanics of cardiac remodelling, TIMPs modulate the activity of MMPs. There are four different TIMPs in vertebrates, TIMP1-4 directly inhibiting MMPs [86]. Like MMPs, their expression tends to be changed during pathological conditions [87]. TIMPs act by forming complexes with the MMPs, rendering the MMPs inactive [86]. 

Therefore, the interplay between MMPs and TIMPs is important for cardiac development and remodelling and like many other proteins in the cardiac ECM, they constitute potential drug targets for conditions such as fibrosis.

## 4. Changes with Age

### 4.1. Fibrosis

Fibrosis is one of the hallmarks of adverse cardiac remodelling—it is observed after trauma, stress and events such as myocardial infarction and hypertension [88,89]. There are three different types of fibrosis: replacement fibrosis, reactive interstitial fibrosis and infiltrative interstitial fibrosis [90]. They all have in common that they can be defined as an increase in ECM-like proteins in the tissue. The three types of fibrosis differ in terms of the precise composition of the ECM proteins secreted, where in the tissue it occurs and what causes it [90]. Reactive interstitial fibrosis refers to the expansion of the cardiac interstitial space with there being no significant death of cardiomyocytes at the same time. This type of fibrosis is highly inflammatory. Replacement fibrosis refers to the deposition of mainly collagen type I into a fibrotic scar in place of cardiomyocytes that have undergone necrosis due to trauma or stress. Infiltrative interstitial fibrosis refers to a build-up of insoluble proteins or glycosphingolipids in the interstitial space in the cardiac tissue [90].

Not surprisingly, fibrosis also makes up the clinical picture of ageing of the human heart. Fibrosis is traditionally seen as a type of scar, and like all scars, it can be considered to be an adaption of the tissue in response to trauma or adverse conditions in the environment. It is an essential part in avoiding devastating outcomes such as rupture of the ventricular wall after myocardial infarction; however, the outcome is not ideal. The resulting tissue, rich in ECM-like proteins such as collagen, is stiffer and less functional than the tissue it replaced [91,92].

### 4.2. Increased Inflammation—The Involvement of Immune Cells and ROS

Many protective occurrences in the body are mixed blessings; fibrosis, which is described above, is one of them, and inflammation is another. Whilst both acute and chronic inflammation, such as that caused by severe acute respiratory syndrome coronavirus 2 (SARS-CoV-2), contributes to adverse cardiac remodelling, as recently reviewed by Gatto et al. [93], we will here focus specifically on how age-associated inflammation contributes to cardiac remodelling. During ageing, the level of inflammation in the heart gradually increases [94,95]. There are several reasons why this happens; one of them is the increased amount of reactive oxygen species (ROS) in the cardiac ECM. As the heart ages, the mitochondria will begin to malfunction, leading to oxidative damage in the cells, which eventually leads to cardiomyocytes undergoing apoptosis [96,97]. Several studies have shown a prevalence of increased ROS in aged hearts of mice and rats, and consequently, it is another hallmark of the aged cardiac ECM [98,99]. Excessive levels of ROS in the tissue will directly affect the ECM, as a number of genes coding for ECM proteins such as collagen and fibronectin are affected by so-called hypoxia-inducible factors (HIFs) [100,101]. When the levels of ROS increase in the tissue or when a state of hypoxia occurs, these HIFs will become active and translocate to the nucleus where they will cause transcription of ECM-related proteins. The result is a direct increase in the deposit of ECM proteins into the matrix. Additionally, HIFs interact with genes coding for enzymes related to the crosslinking and remodelling of the ECM, such as lysyl oxidase and MMPs, leading to an increased level of crosslinking of the ECM [102,103]. 

### 4.3. Altered Protein Levels in the ECM

The proteomic landscape of the cardiac ECM changes with age. However, the findings demonstrating the age-dependent changes are mainly based on studies using male rodents, and in most cases, they only include proteomic changes of the left ventricle. 

**Figure 2:**
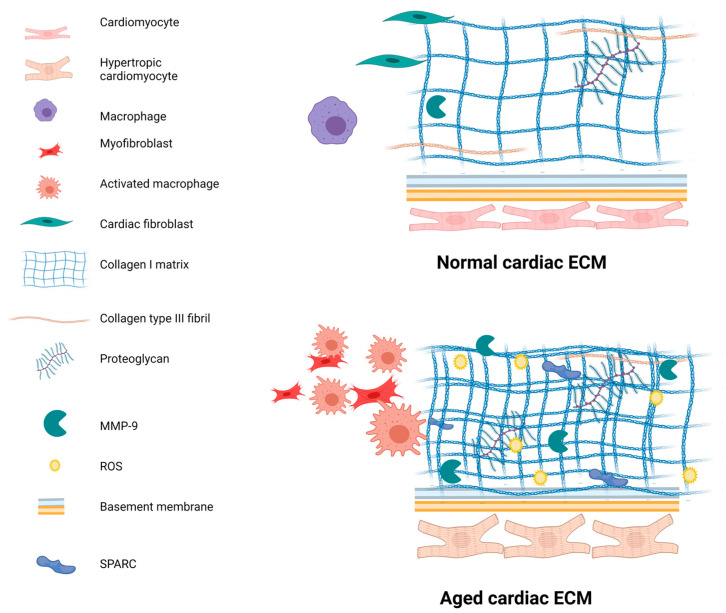
A summary of the proteomic changes of the cardiac ECM with age. Age is associated with changes in the cardiac ECM such as an increase in fibrillar collagen and activation of fibroblasts and macrophages. Created with BioRender.com.

The majority of studies on the composition of the aged cardiac ECM have only included collagens; however, a small number of studies have been published which include other proteins. These studies involved fibronectin, laminin, integrin, mimecan and SPARC, and similarly to studies with a focus on collagen, they have mainly included the left ventricle, with some exceptions [104,105]. Table 2 outlines these proteomic changes with age, including location and species. How ageing changes the cardiac ECM is summarised schematically in Figure 2. Considering how these proteins contribute to normal functioning of the heart, as described previously in Section 2 and Table 1, the relationship between the changes in protein expression and the age-associated decline in cardiac functioning is clear.

In general, the quantity of ECM proteins in cardiac tissue increases with age. However, there are some exceptions. Whilst fibronectin increases in the left ventricles of mice, it decreases in the left ventricles of hamsters and rats [104,106,107]. Some isoforms of laminin and integrins increase with age, and others such as laminin β2 decrease [106,108]. Mimecan decreases in whole hearts and left ventricles in female mice [105,109]. Lastly, one study reported decreased levels of SPARC in the left ventricle of SV129 mice [27]. 

### 4.4. Collagens and SPARC Changes with Age

Of the proteins that make up the cardiac ECM, collagens have undoubtedly been studied the most. For this purpose, hydroxyproline assay and picrosirius red have long been the gold standard. It has been deduced repeatedly that the amount of crosslinked collagen increases with age in the left ventricle in different strains of mice and rats, but also in sheep, Syrian hamsters and humans [27,106,110,111,112,113,114,115].

Whilst the studies on how collagen levels in the atria change with age are few in number, they have been conclusive. Overall collagen content in both atria in male mice and the left atrium in dogs increases with age, and studies on mice revealed an increase in collagen fibrils in the atria with age [113,114,115]. This increase was more evident in the right atrium in both studies involving mice. The study on atria from dogs only included the left atrium, and thus, it is unknown whether this pattern is species general at all. 

In addition to protein levels, gene expression studies indicate that collagen type I and III mRNA decrease in hamsters and rats [104,107]. This decrease in expression was mainly observed in ventricles, but the decrease in type III collagen mRNA also occurs in atria of rats [104,107,110]. There were no significant changes in the collagen type I expression in murine atria and the left ventricles of sheep, and there were no significant changes with age in type III expression in murine atria [111,113]. See Table 3 for an overview of age-related changes in mRNA levels of collagen type I and III and fibronectin.

The reported decrease in collagen type I mRNA levels in cardiac tissue strongly indicates that the age-related increase in collagen type I protein is due to post-translational processes, rather than as a result of increased gene expression. In mice and sheep, studies have revealed an increase in SPARC in the left ventricles [27,111]. As SPARC is involved in collagen fibril deposition, it is plausible that the age-associated increase in SPARC is at least partly responsible for the increase in fibrillar collagen. Indeed, a number of studies have investigated the relationship between SPARC, fibrillar collagen and age. Tissue sections from left ventricles of aged SPARC-null mice sections stained with picrosirius red displayed amounts of fibrillar collagen similar to those of young mice, whilst the aged, wild-type mice possessed the age-associated increase in fibrous collagen [27]. In the same study, scanning electron microscopy further confirmed the lesser extent of fibrillar collagen. A second study on SPARC-null mice concluded that aged, SPARC-null mice exhibit less collagen type III and VI and more collagen type VI than their wild-type counterparts [116]. Due to the effect of SPARC on fibrillar and non-fibrillar collagen types, SPARC may be a potential drug target for CF. However, as the reduction in collagen type III in the aged heart leads to a loss of elasticity, the finding that cardiac tissue from SPARC-null mice exhibits still less collagen type III than wild-type mice signify that caution is required.

**Table 2:** Age-related changes in collagen, fibronectin, laminin, integrin, mimecan and SPARC in atria and ventricles.

	Changes with Age	Species	Anatomical Structure	References
Collagen, overall	Increases	Mice, sheep, rats, humans, dogs	Left ventricle, atria, left atria	[27,106,107,108,109,115,116,117]
Collagen type I	Increases	Humans, sheep	Left ventricle	[27,111,112,113,114,115,116,117,118]
Collagen type III	Decreases in humans	Humans	Left ventricle	[118]
Collagen type IV and VI	Increases	Mice	Left ventricle	[116]
Fibronectin	Increases in mice, decreases in hamsters and rats	Mice, hamsters, rats	Left ventricle, left ventricle and atria in rats	[104,106,109]
Lminin	β1: Increases. Β2: Decreases	Mice	Left ventricle	[108]
Integrin	α1, α5: Increases. β1: Decreases	Mice	Left ventricle	[106]
Mimecan	Decreases	Mice	Whole hearts and left ventricle from females	[105,109]
SPARC	Increases	Sheep, mice	Left ventricle	[27,111]

**Table 3:** Age-related changes in mRNA levels of collagen type I and III and fibronectin in the hearts of rats, Syrian hamsters, mice and sheep.

	Change with Age	Species	Anatomical Structure	References
Collagen type I and III	Decreases in rats and hamsters. No change in sheep. No change in murine atria.	Rats, sheep and Syrian hamsters	Atria and ventricles.	[107,110,111,113]
Fibronectin	Decreases	Rats, hamsters, mice	Atria and ventricles.	[106,107,110]

### 4.5. Changes in MMPs and TIMPs with Age

Several studies have investigated the relationship between MMPs, TIMPs and age. These have mainly been performed on the left ventricle or in plasma, from murine or human tissue. In summary, MMP9 increases with age in the left ventricle, but decreases in plasma [119,120,121,122], MMP28 increases in the LV [120] and MMP1, 2, 3, 7, 8, 12 and 14 increase in the left ventricle [121,122,123]. TIMP3 and 4 decrease with age in tissue from the left ventricle [121]. One study concluded that and TIMP1 increases in plasma from humans [123]. The increase in TIMP1 in plasma has also been found in plasma from mice, in addition to an increase in TIMP 2 and 4 [124]. In contrast, a third study concluded that TIMP1 and 2 decrease with age in plasma from humans [125].

## 5. Conclusions

It is evident that the ECM has a bigger impact on the proper functioning of the heart than thought a few decades ago. As described above, the correct amounts of proteins such as collagen type I and III, decorin and MMPs and TIMPs are essential for structural integrity of the heart, for proper electrical functioning and normal cell–cell communication. Unfortunately, the cardiac ECM remains challenging to study due to the technical difficulties stemming from how tightly bound ECM proteins are in their matrix. As evident from the descriptions of proteome changes with age, there are inconsistencies regarding several different proteins such as collagen and fibronectin. When factoring in the effects of ageing on the cardiac proteomic landscape, it becomes less conclusive still. As highlighted in Table 2 above, some studies have reported different effects of ageing on, e.g., certain TIMPs, even when studying the same species. Moreover, few studies have included the atria as well as the ventricles, and in a similar way, few studies included female animals. As the cardiac ECM is rich in post-translational modifications such as glycosylation and oxidation, there is reason to believe that the extent of these modifications also changes with age. Furthermore, the abundance of ECM proteins such as fibronectin and different collagens varies subject to the precise location within the heart, and it is therefore expected that the changes in the proteomic landscape with ageing affect the structures of the heart in a spatial manner. In this review, the components of the cardiac ECM and the effects of ageing have been discussed as they are understood at the moment. A better understanding of how the cardiac ECM changes with age would benefit the design of safer and more suitable biomaterials and help identify possible drug targets to treat age-associated, ECM-related disorders.

## Data Availability

No new data were created or analysed in this study. Data sharing is not applicable to this article.
